# Floating population diversity as a leading indicator of business turnover imbalance in commercial districts: A spatial panel analysis in Seoul

**DOI:** 10.1371/journal.pone.0332335

**Published:** 2026-02-04

**Authors:** Seongman Jang, Youngsoo An

**Affiliations:** 1 Department of Urban and Regional Development, Mokpo National University, Muan, Republic of Korea; 2 Center for Small Business Insights, Seoul Credit Guarantee Foundation, Seoul, Republic of Korea; Northeast Normal University, CHINA

## Abstract

This study develops a quantitative indicator for the early diagnosis of commercial instability in business openings and closures and proposes a new analytical framework for assessing the stability of commercial districts. To address the limitations of prior research that relied on static measures such as sales or store counts, two complementary approaches were introduced. First, we propose the Commercial Instability Index (CII), a direction-agnostic metric of turnover instability computed from the standardized relative deviation between openings and closures, where larger values indicate greater instability. Second, entropy-based floating population diversity indicators were applied to capture the distribution of visitors by age, time of day, and day of week, as well as their temporal changes. These indicators were tested on quarterly panel data from 1,650 commercial districts in Seoul between the first quarter of 2019 and the fourth quarter of 2024 using panel regression and spatial panel regression models, specifically the spatial autoregressive (SAR) and spatial error model (SEM). The results showed that higher day-of-week diversity consistently reduced the CII, whereas a greater balance in age diversity provided partial mitigation effects. Moreover, the CII revealed significant spatial dependence, indicating that instability in one district could spread to its neighbors. By focusing on the magnitude of instability rather than its direction, and by integrating floating population diversity with spatial dependence, this study advances beyond static approaches. These findings expand the theoretical scope of commercial district research and offer a practical basis for early warning systems and area-based management strategies, thereby contributing to the development of urban policies for resilience and stability of commercial districts.

## Introduction

Commercial districts are core spatial units in cities where consumption, employment, and business activities are concentrated and undergo constant changes through the cyclical process of business openings and closures (hereafter, openings and closures). While such turnover reflects the natural rhythm of urban economies, pronounced volatility in certain areas—such as abnormal clustering of closures or an oversupply of openings—may signal heightened commercial instability. These fluctuations threaten the sustainability of commercial districts and undermine the stability of local economies. However, existing approaches are limited in detecting such structural risks at an early stage. This underscores the need for a new analytical framework to diagnose the dynamic transformations of commercial districts from a structural perspective.

Studies and policies on commercial districts have primarily focused on static outcome indicators, such as sales volume, number of stores, and distribution of business types, which tend to capture only the status at a given point in time. Such approaches are limited in explaining the structural instability that accumulates through repeated openings and closures, and often remain a descriptive interpretation of short-term changes [1–3]. Moreover, analyses of floating populations have largely relied on aggregate measures such as size or density, failing to adequately reflect compositional biases in age, time-of-day, or day-of-week composition [4–6]. Consequently, existing analyses have been insufficient for detecting risks in commercial districts in advance or for functioning as an early warning system.

This study addresses these gaps by examining the magnitude of instability and by integrating composition with space. First, we propose the Commercial Instability Index (CII), a direction-agnostic outcome that quantifies the magnitude of turnover instability through the standardized relative deviation between openings and closures (larger values indicate greater instability). The formal definition appears in the construction of indices. Second, we introduced the Shannon entropy index (hereafter, the entropy index) to quantify floating population diversity based on age, time-of-day, and day-of-week composition, and we incorporated its temporal variation into the analysis. Third, we applied these indicators to panel and spatial panel regression models to empirically examine the relationship between floating population diversity and the CII. This study moves beyond static outcome-based approaches and proposes a new analytical framework for detecting structural risks in commercial districts at an early stage.

To achieve these objectives, this study posed two central research questions: First, can the diversity of the floating population attributes and their changes serve as early signals for the CII in commercial districts? Second, is commercial instability confined to individual commercial districts, or does it exhibit spatial dependence by spilling over to adjacent districts?

The remainder of this paper is organized as follows: We review the literature on commercial-district instability, floating population diversity, and the entropy index, and present the theoretical background. The next section describes the data, construction of variables, and analytical methods, including the panel and spatial panel regression models. The subsequent sections report the empirical results and conclude the study by synthesizing the main findings and discussing policy implications.

### Literature review

#### Dynamics of commercial districts and business openings and closures.

Commercial districts are not merely fixed spaces in which stores are concentrated. Rather, they are inherently dynamic spatial units in which consumption, employment, and business activities are clustered and continuously reorganized in response to changing socioeconomic conditions. Gu [7] described commercial areas as the core of urban vitality, noting that their boundaries and functions are constantly adjusted by economic flow, policy shifts, and transformations in urban structures. Similarly, Minner and Shi [8] highlighted that remodeling activities and landscape changes along commercial strips signal broader economic and social restructuring, emphasizing that commercial districts are continually reconstituted within a temporal context. These discussions suggest that commercial districts should be understood not as static measures of spatial density, but as dynamic entities that accommodate and interact with diverse urban flows.

This perspective has been consistently emphasized not only in recent empirical studies but also in long-standing urban theories. Through the *concentric zone model*, Burgess [9] explained urban growth and structure by showing that the central city and transitional zones are dynamic spaces characterized by constant change and heterogeneity. Transitional zones are areas where residential, commercial, and industrial functions coexist with high turnover rates and repeated openings and closures, making them unstable and cyclical in nature. Jacobs [10] regarded cross-industry interactions and *emergent order* as key elements of urban vitality, emphasizing that openings and closures function as integral components of urban ecosystems. In analyzing the employment contributions of small businesses in the United States, Birch [11] noted that openings and closures constitute a natural cycle of urban economies in which job creation and destruction occur simultaneously within commercial districts. These classical discussions reinforce the view that commercial districts are not fixed spaces but organic entities reflecting economic dynamism within the broader urban structure.

Building on this theoretical foundation, efforts to quantify the variability of commercial districts have expanded through a range of empirical studies, in which openings and closures have been widely used as indicators of change. These extend beyond simple counts of stores and serve as tools for assessing the sensitivity and restructuring potential of commercial districts in response to external shocks and local characteristics. For example, Bartik et al. [12] conducted a large-scale survey of small businesses across the United States to quantitatively analyze the impact of the pandemic on closure rates and reopening prospects. Minner and Shi [8] spatially tracked store replacement and remodeling activities along commercial strips, showing the extent to which variability is closely linked to the reorganization of the urban commercial environment. Similarly, Kim and An [13] analyzed the dynamic changes in alley-type commercial districts using store diversity and density, while Park et al. [14] classified districts into growth and decline types based on sales flows and store continuity, highlighting the limitations of static indicators such as opening and closure rates. Although approaches that capture openings, closures, and store continuity have become central tools for commercial district analysis because of their suitability for time series and quantitative assessments, most studies have focused on the occurrence of these events themselves, with relatively less attention to the structural implications of commercial instability arising from differences between openings and closures.

This study focuses not on the total volume of openings and closures but on the magnitude of commercial instability they generate. Even when openings are numerous, the concentration of closures in specific periods or areas can render commercial districts unstable. Artz et al. [15] argued that in urban areas, where openings and closures occur more frequently than in rural areas, structural dynamics cannot be fully explained by aggregate indicators alone. Park et al. [14] classified commercial districts based on sales flows and store continuity, highlighting the limitations of static indicators such as opening and closure rates. These discussions suggest that relative proportions, timing, and spatial distributions that produce instability offer more critical criteria than simple counts for assessing the health of commercial districts. Accordingly, we emphasize structural signals of instability—situations wherein the balance between openings and closures breaks down—and link these dynamics to commercial-district stability outcomes. To do so, we propose an interpretive framework that extends beyond conventional static approaches.

#### Concept and measurement of floating population diversity.

The floating population is not merely the volume of pedestrian movement but also an indicator that reveals how cities function, exposing temporal and spatial density changes that cannot be explained by the resident population [16]. It reflects patterns of urban space use and perception, serving as both the agents of consumption, employment, transportation, and service utilization and as the demand base for commercial districts. Byun and Seo [17] indicated that the vitality of downtown commercial districts was directly linked to daytime population density, emphasizing the critical role of the floating population in determining the value and survival of commercial spaces. Accordingly, the floating population was established as an essential variable for capturing both economic flows and administrative demand and was positioned as a core variable in the analysis of commercial districts in this study.

Despite their importance, research on floating populations has largely relied on aggregate indicators such as pedestrian counts or density. These approaches have limited capacity to capture compositional biases, diversity, and long-term dynamics, as reported in studies in Seoul and other urban contexts [4–6]. While useful for explaining the overall vitality and inflows, such measures fail to reveal the structural risks within commercial districts.

Analyses of floating populations should focus on compositional diversity rather than on scale alone. Even when inflows are of equal size, a strong concentration in particular age groups or specific times and days can render commercial districts highly vulnerable to shifts in only a subset of users. Conversely, when users with different purposes and time schedules are more evenly distributed, the demand base becomes diversified and buffered, reducing the sensitivity to external shocks. Jacobs [10] argued that lively streets are sustained when used for multiple purposes and when time frames overlap, whereas Ozer and Kubat [18] showed that the composition and intensity of pedestrian flows vary by day and group characteristics, and that such concentration can constrain street activity. Similarly, Byun and Seo [17] demonstrated through attribute-based disaggregation that even with the same aggregate volume, compositional biases shaped the stability and quality of spatial use. Taken together, these discussions underscore that quantitatively measuring the floating population diversity is central to assessing commercial-district stability and diagnosing instability risk.

Attempts to quantify floating population diversity have existed, but they have often relied on simple measures, such as means, variances, ratios, or single conversion factors. Such approaches fail to fully capture the complexity and balance of categorical structures. For instance, Jiménez et al. [19] classified pedestrians by type to highlight design and management needs; however, their analysis remained conceptual and did not quantitatively reveal inter-group distributional biases or interaction structures. Likewise, Galiza and Ferreira’s [20] *Standard Pedestrian Equivalent* approach integrated group heterogeneity into a single coefficient, which was useful for capacity estimation, but limited to explaining distributional imbalance (compositional bias) across categories. In contrast, Jiang et al. [21] analyzed the origin–destination distributions of taxi trips in New York City using entropy, effectively capturing the spatial diversity and responses to external shocks that could not be identified through simple aggregate measures. These findings reinforce the utility and necessity of entropy indices, reflecting both the complexity and the balance of categorical distributions.

In practice, information-theoretic measures such as Shannon entropy have been applied across diverse fields, including urban planning, transportation, and spatial analysis. Cervero and Kockelman [22] identified land-use mix (LUM) as a key factor influencing travel demand, together with density and design. Frank et al. [23] empirically examined the relationship between walkability and residents’ physical activity using an LUM index derived from Shannon entropy. Yeh and Li [24] applied entropy to quantify the spatial balance of land use and to diagnose urban sprawl, whereas Altieri et al. [25] extended the measure to assess spatial heterogeneity across ecological, geographical, and planning contexts. Jiang et al. [18] also used entropy to analyze the origin–destination distributions of taxi trips in New York City, quantifying the spatial diversity and pre- and post-shock changes that could not be revealed by simple aggregate measures. Collectively, these studies support the view that entropy indices are valid and versatile tools for urban spatial analyses in areas such as land use, transportation, and spatial networks.

This study extends the use of entropy indices previously applied in urban planning, transportation, and spatial network analysis to measure the floating population diversity. Specifically, Shannon entropy was applied to categorical attributes (age, time of day, and day of the week) to quantify compositional diversity rather than mere aggregate volume. This approach enables the detection of compositional biases that arise when the demand is concentrated in particular groups or time segments, thereby complementing the limitations of aggregate-based measures. The resulting metric provides a new analytical framework for diagnosing the structural characteristics and potential risks of floating populations in the context of commercial district analysis and urban planning. Furthermore, when combined with the CII—which quantifies the magnitude of instability in business openings and closures—this approach provides a foundation for a comprehensive understanding of commercial-district dynamics and stability.

#### Limitations of existing commercial district analyses and prediction models.

Most existing analyses of commercial districts remain cross-sectional, relying on static indicators, such as the number of stores, sales volume, and business composition at a single point in time. As such, approaches focus on changes in store counts or the proportion of business types; they fail to adequately capture temporal patterns of change, the cyclical nature of openings and closures, or structural instability within districts. Lee et al. [1] applied a deep learning model to predict the number of stores and average sales in commercial districts and noted that many analyses and forecasts depend on single-period data, thereby limiting their ability to explain long-term dynamics. This concern was raised decades earlier: Wegener [2], in his analysis of urban and regional systems, criticized single-period and single-sector static approaches for overlooking complex spatial–temporal interactions and emphasized the need for dynamic and integrated analytical frameworks.

These limitations are not confined to simple descriptive analyses but are repeatedly observed in forecasting models. Existing prediction models for commercial districts have mainly focused on estimating changes in outcome indicators, such as sales volume, number of stores, and land demand. For example, Batista and Silva et al. [26] proposed a model to estimate industrial and commercial land demand based on economic forecasts; however, they did not analyze the structural drivers of instability that underpin such demand. Glaeser and Gottlieb [27] also emphasized the economic drivers of urban growth and productivity change but focused less on structural disparities or the mechanisms of spatial instability across regions. Similarly, the urban land demand forecasting framework suggested by Waddell and Moore [28] demonstrated strengths in market-based estimation but did not reflect structural factors such as compositional biases in population and business types within commercial districts or the cyclical patterns of openings and closures. Ultimately, such outcome-oriented approaches lack explanatory power for why particular commercial districts become unstable, and they fail to integrate multiple factors, including changes in floating-population composition, rhythms of openings and closures, and inter-district interactions.

This lack of structural explanatory power is evident in the treatment of the spatial and structural contexts. Existing analyses have generally regarded individual commercial districts as independent units without adequately accounting for interactions with adjacent districts or spatial spillover effects. However, urban and regional systems are characterized by blurred boundaries between districts and the spatial transmission of activation or decline across neighboring areas. Such interdependencies are difficult to capture through static figures such as simple changes in store counts or sales. Batty [3] conceptualized urban systems as “connected subsystems,” emphasizing the need for integrated models that simultaneously consider spatial structures and flows. This integrative perspective was further developed by Wegener [2], who explained that urban and regional systems evolve through interactions among multiple markets (employment, housing, commerce, land, etc.) and warned that neglecting such feedback risks overlooking critical structural information. Building on this theoretical foundation, Piovani et al. [29] empirically demonstrated the limitations of treating commercial districts in isolation by analyzing retail locations and spatial linkages using the percolation theory and spatial interaction models.

To address these limitations, this study designed an analytical framework in three key directions.

First, it operationalizes the magnitude of instability between openings and closures using the CII. Second, it introduces floating-population compositional diversity—age, time-of-day, and day-of-week—as explanatory variables for the CII. Third, it employs quarterly panel data and spatial panel regression models to capture the temporal dynamics and spatial spillover effects jointly. This approach enabled the dynamic identification of compositional biases and instability, rather than relying on aggregate changes alone, thereby allowing for a more comprehensive diagnosis of both the stability and variability of commercial districts. Most importantly, by incorporating inter-district interactions, this study advances an expanded analytical perspective that integrates dynamism, structure, and spatiality, offering a new framework that clearly distinguishes it from the existing research.

Building on classic urban-vitality theories—which emphasize multi-use, overlapping time frames, and cross-group mixing as stabilizers of street-level activity—we translate these ideas to commercial districts: Diversity in when (day/time) and who (age) uses a district broadens the effective demand base and dampens variance in openings/closures.

We posit that floating-population diversity stabilizes commercial demand through portfolio-like risk spreading across user groups and time slots. When visits are more evenly distributed across weekdays/weekends, hours, and age groups, adverse shocks to any single segment are partially offset by others, lowering the CII. Conversely, temporal or demographic concentration heightens exposure to idiosyncratic shocks and increases the CII. This mechanism motivates our empirical tests in the panel and spatial-panel frameworks.

## Data and methodology

### Data sources and variable construction

**Fig 1:**
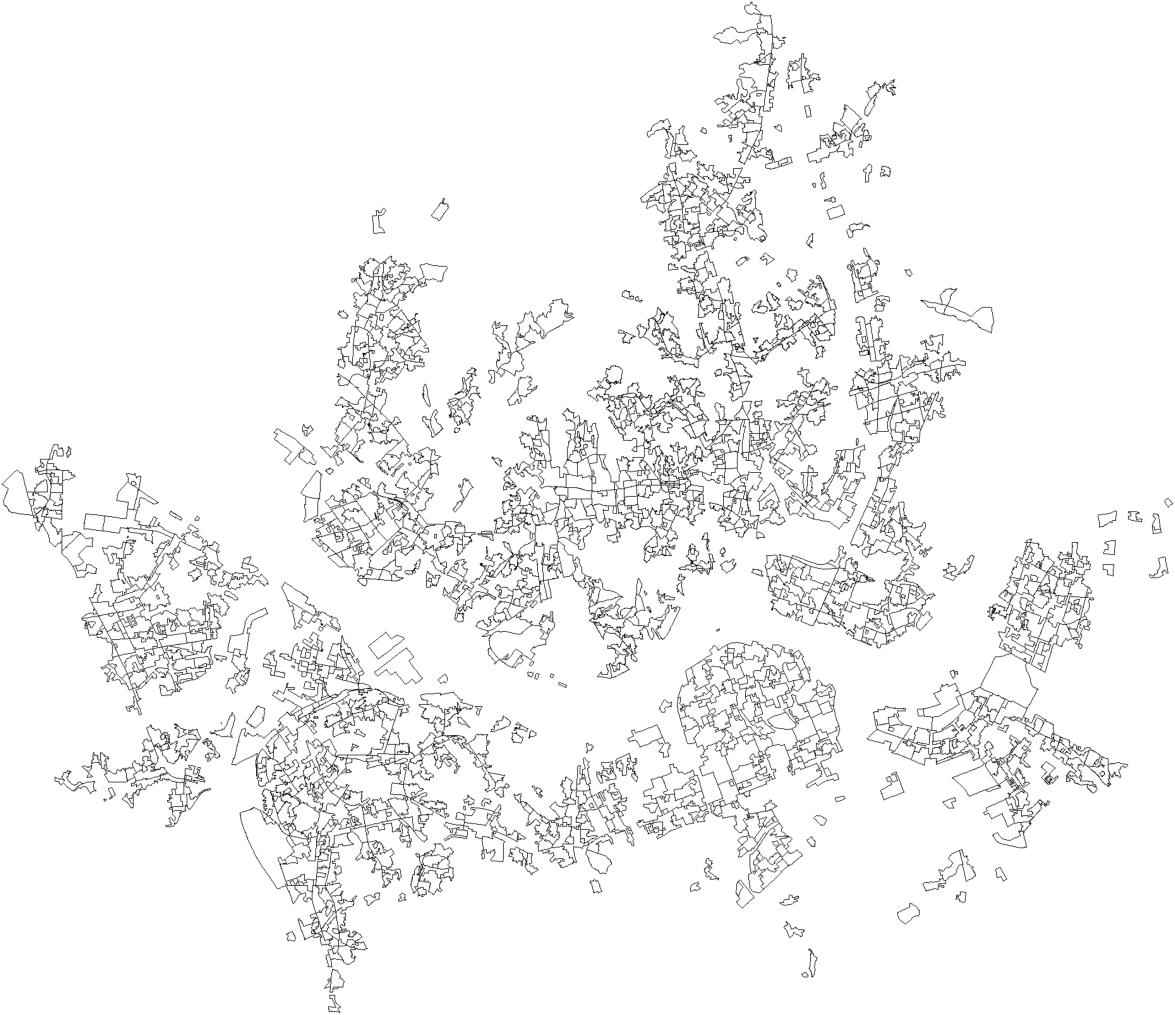
Study area and commercial district boundaries. Note: The map shows the spatial extent of Seoul’s commercial districts used in the analysis. Boundary data source: Seoul Commercial Area Analysis Service (Area–Commercial District boundary), provided by the Seoul Credit Guarantee Foundation; copyright Seoul Metropolitan Government, licensed under KOGL Type 1 (Attribution; commercial use and modification permitted). Figure created by the authors; no proprietary basemap used.

The empirical analysis in this study focuses on major commercial districts in Seoul that are primarily composed of small businesses, defined here as storefront establishments across the city’s 100 daily-life sectors—retail (36), food-and-beverage (12), lodging (2), and services (50)—as classified by the Seoul Commercial District Analysis Service. The unit of analysis was based on commercial district boundaries provided by the Seoul Credit Guarantee Foundation (Shapefile), encompassing 1,650 districts. We did not distinguish effects by industry. Instead, we pooled all storefront sectors and aggregated quarterly openings and closures within each district, applying no sectoral weighting in constructing the outcome and regressors. These spatial units are more finely delineated than administrative neighborhoods (dong), allowing for a more precise observation of variations in openings and closures as well as changes in the floating population across local areas. Fig 1 shows the spatial distribution of the commercial districts throughout Seoul and provides an intuitive representation of their densities and spatial characteristics.

The data on openings and closures and the floating population used in this study were obtained from the Seoul Commercial District Analysis Service. The openings and closures dataset includes the number of new business openings and closures per quarter, whereas the floating population dataset provides counts of movement disaggregated by gender, age, day of the week, and time of day. Openings and closures are compiled from business-registration records released quarterly. The dataset provides event counts keyed to commercial-district identifiers; we aligned these identifiers with the Seoul Credit Guarantee Foundation commercial-district shapefile and aggregated openings and closures at the district–quarter level. Floating-population counts originate from the Seoul–KT “living population” product, provided on 10-meter road segments; we spatially aggregated these segments to commercial-district polygons for each quarter.

The study period spanned 24 quarters, from the first quarter of 2019 to the fourth quarter of 2024. The dependent variable, the CII ($I_{t}$), was calculated from the second quarter of 2019 to the fourth quarter of 2024. This reflects the fact that the diversity indices of the floating population attributes (age, time of day, and day of the week) lagged by one-quarter in the analysis. Specifically, the diversity index ($H_{t}^{\alpha }$) was computed using the previous quarter’s data to account for temporal lags. The change in diversity ($\Delta H_{t}^{\alpha }$) was then calculated as the difference between the diversity value for each quarter and that of the preceding quarter.

### Construction of indices

In this study, three indices are constructed to quantitatively capture the structural characteristics and temporal dynamics of commercial districts. First, the CII measures the numerical discrepancy between business openings and closures, with larger deviations indicating greater structural instability. Second, the diversity index evaluates how evenly the floating population is distributed across age groups, days of the week, and times of the day; higher values denote a more balanced distribution across categories. Third, the change in the diversity index measures the increase or decrease in diversity relative to the previous quarter, thereby capturing both the direction and magnitude of temporal shifts in population composition.

#### Commercial instability index.

We operationalize instability using the CII, a direction-agnostic measure of turnover instability based on the standardized relative deviation between openings and closures. This index is defined as follows:


$$I_{t}=\frac{|Z_{open,t}-Z_{close,t}|}{\sqrt{2}}$$ (1)

where $Z_{open,t}$ and $Z_{close,t}$ are the standardized (z-score) values of the numbers of business openings and closures, respectively, for period t. The denominator $\sqrt{\text{2}}$ is applied because the variance of the difference between two independent standardized scores equals 2; dividing by $\sqrt{\text{2}}$ standardizes this difference to the unit variance, thereby facilitating comparability across commercial districts. In practical terms, lower values (near zero) indicate greater stability, whereas higher values indicate greater instability in openings and closures. We ignore the sign because our early-warning objective targets risk magnitude, not direction, and we treat either case wherein openings exceed closures or closures exceed openings as elevated instability.

#### Floating population diversity index.

The structural diversity of the floating population was measured using an entropy index based on the Shannon information theory [30]. The entropy index quantifies the evenness of the distribution across categories, with higher values indicating a more balanced distribution and thus greater diversity. In this study, the floating population within each commercial district was classified according to the age group, day of the week, and time of day. This index is defined as follows:


$$H_{t}^{\alpha }=-\sum_{i=1}^{n}p_{i}^{\alpha }{𝐥𝐨𝐠}_{2}\left(p_{i}^{\alpha }\right),\;\sum_{i}p_{i}^{\alpha }=1$$ (2)

where $p_{i}^{\alpha }$ denotes the proportion of floating population in category i of attribute α (age group, day of the week, or time of day) at time t, and n represents the total number of categories for that attribute. We use the base-2 logarithm, so $\text{0}\leq H_{t}^{\alpha }\leq {\text{log}}_{\text{2}}(n)$. By convention, terms with $p_{i}^{\alpha }=0$ are set to zero (i.e., $\text{0}\cdot {\text{log}}_{\text{2}}\text{0}\;:=\text{0}$). The categorical classifications are as follows:

Age groups: 10s, 20s, 30s, 40s, 50s, 60s and above (n = 6)

Days of the week: Monday–Sunday (n=7)

Time periods: 00:00–06:00, 06:00–11:00, 11:00–14:00, 14:00–17:00, 17:00–21:00, 21:00–24:00 (n=6)

The entropy index has been widely applied in urban planning and studies to measure land-use heterogeneity, commercial facility diversity, and demographic composition [21,25,31]. In this study, it was used to quantify the structural diversity of the floating populations within each commercial district.

#### Change in the floating population diversity index.

The change in the floating population diversity index measures the extent to which the diversity level in a given quarter differs from that of the previous quarter. This indicator was designed to quantify structural changes over time that cannot be fully captured by the diversity index alone. In this study, it was calculated as follows:


$$\Delta H_{t}^{\alpha }=H_{t}^{\alpha }-H_{t-1}^{\alpha }$$ (3)

where $H_{t}^{\alpha }$ and $H_{t-\text{1}}^{\alpha }$ denote the diversity index values of attribute α (age group, day of the week, or time of day) at time t and t−1, respectively. A positive value of $\Delta H_{t}^{\alpha }$ indicates an increase in the diversity of the floating population for the given attribute compared to the previous quarter, whereas a negative value indicates a decrease.

This index captures not only the static level of diversity but also its temporal variation, thereby offering a more precise and in-depth analytical perspective. In particular, its ability to detect both the direction and magnitude of change in a time-series analysis simultaneously makes it particularly useful. By considering both the absolute level and rate of change in diversity, the index enables a more comprehensive and systematic diagnosis of the structural stability and variability of commercial districts.

### Analytical models

#### Overview of analytical models.

This study assembles a quarterly panel of 1,650 commercial districts in Seoul from the first quarter of 2019 to the fourth quarter of 2024. For the spatial panel estimations (Spatial Autoregressive Model [SAR]/ Spatial Error Model [SEM]), we restricted the sample to districts with complete 23-quarter histories after applying a one-quarter lag. The panel structure provides repeated observations for the same districts, enabling us to control for time-invariant district heterogeneity via district fixed effects and exploit within-district quarterly variations.

However, commercial districts are geographically contiguous, and both the floating population and business openings and closures are likely to be spatially diffused. When such spatial dependence exists, conventional regression models may yield biased and inconsistent estimates [32]. Accordingly, this study first applies traditional panel regression models (pooled ordinary least squares [OLS] and fixed effects) and then estimates spatial panel regression models, specifically the Spatial Autoregressive Model (SAR) and the Spatial Error Model (SEM), to correct for spatial dependence.

#### Panel regression models.

Panel regression models utilize repeated observations of the same units, allowing unit-specific heterogeneity and temporal dynamics to be considered jointly [33]. In this study, the dependent variable is the CII, and the key regressors are the one-quarter lagged diversity index of floating population attributes (age group, time of day, and day of the week) and the change in diversity. First, we estimate a pooled ordinary least squares (OLS) model that does not control for unit-specific effects. Although pooled OLS treats the data as a single cross-section and is simple to interpret, the estimates can be biased when unobserved unit-specific heterogeneity is correlated with the regressors. To address this concern, we estimate a fixed-effects (within) model that removes time-invariant unit-specific components. While the fixed-effects model mitigates the endogeneity arising from the correlation between the regressors and unobserved heterogeneity, it cannot identify the effects of time-invariant variables [34].

In this study, we estimated both models in parallel and compared the results to assess the influence of the time-invariant characteristics across commercial districts on the estimation outcomes. The dataset was structured as a district-by-quarter panel, with as the dependent variable and the lagged diversity index of the floating population attributes, together with changes in diversity, as the main explanatory variables.


$$I_{t}=\beta_{0}+\beta_{1}H_{t-1}^{age}+\beta_{2}H_{t-1}^{time}+\beta_{3}H_{t-1}^{day}+\beta_{4}\Delta H_{t-1}^{age}+\beta_{5}\Delta H_{t-1}^{time}+\beta_{6}\Delta H_{t-1}^{day}+u_{i}+\epsilon_{it}$$ (4)

where $u_{i}$ denotes the fixed effect capturing time-invariant, district-specific heterogeneity, and $\epsilon_{it}$ represents the idiosyncratic error term. These fixed effects absorb unobserved district factors that do not vary over time, and the identification relies on within-district quarterly variations.

However, commercial districts are geographically adjacent, and the effects of openings, closures, and floating population activities can easily spread across spaces. Traditional panel regression models can produce biased and inconsistent estimates in the presence of spatial dependence. To address this issue, we apply spatial panel regression models.

#### Spatial panel regression models.

Commercial districts are organized as spatially adjacent units, and both floating population activities and openings and closures can readily spill over into neighboring areas. In the presence of spatial dependence, traditional panel regression models may yield biased and inconsistent estimates [32,35]. To address this issue, this study employs two types of spatial panel regression models: SAR and SEM.

The SAR model incorporates a spatial lag term to account for the spatial autocorrelation in the dependent variable. The model is expressed as follows:


y=ρWy+Xβ+μ (5)

where ρ represents the strength of spatial dependence, and W denotes the predefined spatial weight matrix.

In contrast, the SEM accounts for the spatial correlation in the error terms and is expressed as follows:


y=Xβ+μ,           μ=λWμ+ϵ (6)

where λ represents the strength of the spatial error dependence.

The SAR and SEM models were estimated using a panel data structure that incorporated both district (cross-sectional unit) and quarter (time) dimensions. To control for the time-invariant characteristics of each district, a fixed-effects specification with district fixed effects was applied, while spatial dependence was modeled using the SAR/SEM structure. The spatial weight matrix W is a row-standardized k-nearest neighbor (k = 4) inverse-distance matrix constructed from the district centroids. Both models were estimated using maximum likelihood.

The necessity of applying spatial models was verified using the Lagrange multiplier (LM) test with data from the most recent quarter. Conducting the test for all quarters could lead to redundancy and reduced efficiency. Therefore, the diagnosis was performed during the latest available period. The results indicate that both spatial autocorrelation in the dependent variable (LM-Lag) and spatial autocorrelation in the error term (LM-Error) were statistically significant, and the robust LM tests confirmed the same pattern.

## Results

### Descriptive statistics

This study employed the CII as the dependent variable, along with the diversity indices of the floating population by attribute in the previous quarter (t–1)— the age, time-of-day, and day-of-week diversity indices —as the main independent variables. In addition, quarter-to-quarter changes in each diversity index (age, time of day, and day of the week) were incorporated into the analysis. Table 1 presents the mean, standard deviation, and minimum and maximum values of all variables.

**Table 1:** Descriptive statistics of variables.

Variable	Mean	Std. Dev.	Min	Max
Commercial instability index (CII)	0.132	0.259	0.000	11.714
Age diversity index	2.507	0.072	0.000	2.584
Time-of-day diversity index	2.510	0.048	1.053	2.569
Day-of-week diversity index	2.803	0.018	0.997	2.807
Change in age diversity	0.000^†^	0.022	−2.532	0.997
Change in time-of-day diversity	0.000^†^	0.015	−1.893	0.346
Change in day-of-week diversity	0.000^†^	0.015	−2.606	0.764

Note: Reported values are means, standard deviations (Std. Dev.), minimum (Min), and maximum (Max) values for all variables over the study period. ^†^: indicates a negative value rounding to zero.

The mean value of the CII was 0.132, indicating a relatively low level on average; however, the maximum value reached 11.714, suggesting that some commercial districts experienced very high instability. The mean values of age, time of day, and day of the week diversity indices were 2.507, 2.510, and 2.803, respectively. The standard deviation was lowest for the day-of-week diversity index (0.018), implying the smallest variability across districts. The mean values of the change measures were all very close to zero, but their magnitudes differed across attributes, with the standard deviation being the highest for the change in age diversity (0.022). This indicates that the variability in the age composition was relatively greater than that in the time-of-day or day-of-week composition. These descriptive results provide a critical foundation for interpreting the relationship between the CII and diversity indices in subsequent analyses.

**Fig 2:**
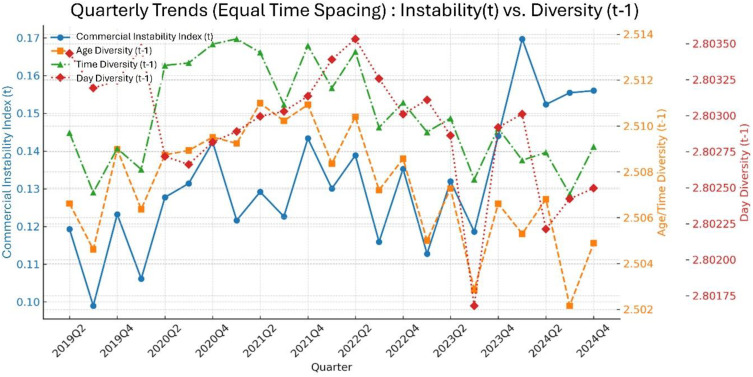
Quarterly trends in the CII and diversity indices. Note: Reported values are quarterly averages for the CII and three diversity indices (age, time of day, and day of the week)*.*

Fig 2 presents the time-series trends of the CII and diversity indices of floating population attributes (age, time of day, and day of the week), measured based on the previous quarter (t–1), from the second quarter of 2019 to the fourth quarter of 2024. The CII fluctuated across quarters during the study period, but showed a clear increase from the third quarter of 2023 onward, remaining at relatively higher levels compared to earlier periods. This pattern indicates that district-level instability in openings and closures has intensified in recent years.

Age and time-of-day diversity indices remained relatively high from 2020 to the end of 2021. Since 2021, both indices have gradually declined, reaching their lowest levels by the third quarter of 2024. Throughout the study period, the two indices exhibited highly similar fluctuation patterns, suggesting that the demographic composition and usage behaviors within commercial districts might have been influenced by common factors. In contrast, the day-of-week diversity index displayed a very narrow range and minimal variation, maintaining a stable level throughout the entire period. Unlike the CII, this index does not exhibit any pronounced co-movement, indicating its limited association with short-term fluctuations.

**Fig 3:**
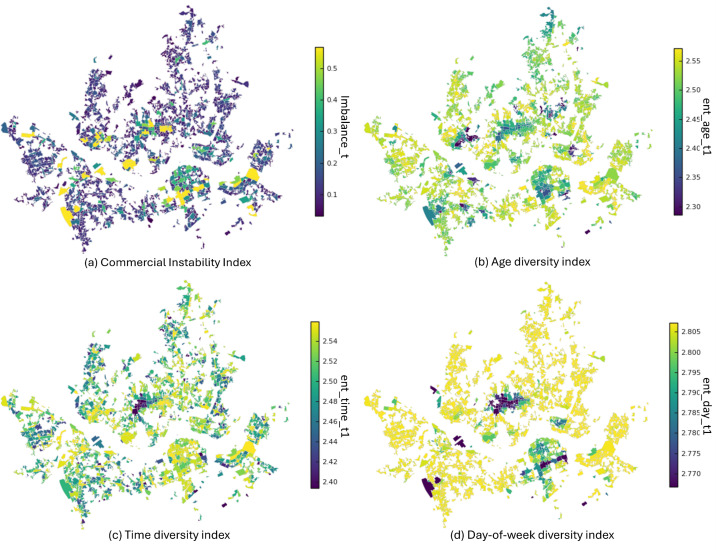
Spatial distribution of the CII and three diversity indices (age, time of day, and day of the week). Note: Darker colors represent lower index values, while lighter colors represent higher values. All values are averaged over the study period. Boundary data source: Seoul Commercial Area Analysis Service (Area–Commercial District boundary), provided by the Seoul Credit Guarantee Foundation; copyright Seoul Metropolitan Government, licensed under KOGL Type 1 (Attribution; commercial use and modification permitted). Figure created by the authors; no proprietary basemap used.

Fig 3 illustrates the average values of the CII and three floating population diversity indices (age, time of day, and day of the week) across the commercial districts during the study period. Each index was visualized using color intervals to represent the relative magnitudes, with lighter and darker colors indicating higher and lower values, respectively.

The CII was relatively high in large-scale commercial and office clusters located in the central and southern parts of the city, whereas it was lower in peripheral residential districts with limited commercial functions. Districts with higher values are typically characterized by strong concentrations of business and office activities, whereas lower values are found in areas dominated by residential functions or with a smaller-scale commercial presence. This indicates that differences in the function and scale of commercial districts shape the composition of the floating population, which in turn manifests as a variation in CII levels.

Lower levels of age diversity were observed in two types of areas: (1) university-centered districts, where young adults in their early 20s are highly concentrated; and (2) major business districts, where floating populations are dominated by office workers in their 30s–50s. By contrast, residential areas and small- to medium-sized commercial districts showed relatively higher levels, reflecting a more balanced age composition.

The time-of-day diversity index was lowest in business- and tourism-oriented districts, where activities were heavily concentrated during daytime hours. In contrast, districts with mixed functions—residential, commercial, business, and cultural—displayed higher values owing to a more even distribution of floating populations across different time periods.

The day-of-week diversity index remained relatively high across most districts. However, major employment and business hubs recorded lower values, reflecting a strong concentration of activities on weekdays. In comparison, peripheral districts and adjacent residential areas exhibited smaller differences between weekdays and weekends, resulting in a higher day-of-week diversity.

### Panel regression results

This section examines the relationship between the diversity indices of the floating population (age, time of day, and day of the week) and the CII using baseline panel regression models (pooled OLS and fixed effects). The Pooled OLS model treats all data as a single cross-section without controlling for district-specific characteristics, whereas the fixed-effects model removes the time-invariant characteristics of each commercial district to capture temporal variation. By applying both models, we compared the extent to which district-specific characteristics affected the results. These findings are summarized in Table 2.

**Table 2:** Panel regression results (pooled OLS and fixed effects).

Variable	Pooled OLS			Fixed Effects		
	Coef.	Std. Err.	p-value	Coef.	Std. Err.	p-value
Intercept	4.937	0.219	<0.001			
Age diversity index	−0.054	0.020	0.007	−0.031	0.050	0.528
Time-of-day diversity index	0.561	0.029	<0.001	0.011	0.056	0.848
Day-of-week diversity index	−2.168	0.087	<0.001	−0.044	0.125	0.722
Change in age diversity	−0.108	0.075	0.150	−0.090	0.057	0.113
Change in time-of-day diversity	0.641	0.123	<0.001	−0.080	0.093	0.388
Change in day-of-week diversity	−0.765	0.140	<0.001	0.143	0.107	0.182
N	37,935			37,935		
$R^{\text{2}}$($Adj.\;R^{\text{2}}$) | Within $R^{\text{2}}$	0.024 (0.023)			0.000		

Note: The dependent variable is the CII. Standard errors are reported in the Std. Err. column.

According to the pooled OLS results (Table 2), the day-of-week diversity index (p < 0.001) has a negative coefficient, indicating that a more even distribution between weekdays and weekends is associated with lower CII levels (i.e., greater stability). Similarly, the change in day-of-week diversity (p < 0.001) also exhibits a negative coefficient, suggesting that shifts toward a more balanced distribution across days are associated with reductions in the CII.

According to the fixed effects results (see Table 2), none of the coefficients were statistically significant, and the explanatory power was extremely low (Within R² ≈ 0.0001). The fixed-effects model removes the time-invariant characteristics of each commercial district that cannot be controlled for in the pooled OLS, thereby estimating the effects based on within-district temporal variation. These results indicate that within-district variation alone does not sufficiently explain the relationship between the diversity indices of the floating population and the CII. However, because this model does not account for interactions with neighboring districts, the next section discusses the application of spatial panel regression models (SAR and SEM) to address this limitation.

### Spatial panel regression results

Spatial panel regression models are applied to address the limitations identified in the fixed-effects model, namely, the inability to account for spatial dependence across commercial districts. Spatial panel models are divided into two types: the SAR model, which captures spatial dependence when the dependent variable is directly influenced by neighboring values, and the SEM, which incorporates spatial correlation in the error terms arising from omitted variables that affect the dependent variable. The following subsections present the estimation results, interpretations, and model comparisons to examine the spatial dependence characteristics (see Table 3).

**Table 3:** Spatial panel regression results (SAR and SEM models).

Variable	SAR			SEM		
	Coef.	Std. Err.	p-value	Coef.	Std. Err.	p-value
Age diversity index	−0.0606	0.0529	0.252	−0.0697	0.0552	0.207
Time-of-day diversity index	0.0066	0.0588	0.911	0.0155	0.0593	0.794
Day-of-week diversity index	−1.7344	0.3621	<0.001	−1.5858	0.374	<0.001
Change in age diversity	−0.1253	0.0732	0.087	−0.1256	0.0766	0.101
Change in time-of-day diversity	−0.107	0.0993	0.281	−0.0971	0.1007	0.335
Change in day-of-week diversity	0.3523	0.4422	0.426	0.2754	0.4585	0.548
Spatial parameter (ρ/ λ)	0.1094	0.0068	<0.001	0.1079	0.0068	<0.001
N	37,935			37,935		
Pseudo R²	0.5335			0.5295		

Note. Dependent variable: CII. Maximum-likelihood spatial panel with district fixed effects; W = row-standardized k-nearest-neighbors (k = 4) inverse-distance. Std. Err. reported; spatial parameter ρ (SAR)/ λ (SEM). Pseudo R² = 1 − RSS/TSS. Balanced 23-quarter design; final N reflects minimal exclusions due to missing values. SAR, Spatial autoregressive model; SEM, Spatial error model.

In the SAR model (Table 3), the coefficient of the day-of-week diversity index (p < 0.001) was negative, indicating that a more balanced distribution of floating populations between weekdays and weekends was associated with a lower CII (i.e., greater stability). Age and time-of-day diversity indices were not significantly different. Among the change variables, the change in age diversity (p < 0.1) showed a negative effect, suggesting that adjustments toward a more balanced age composition are linked to reductions in the CII. The spatial autoregressive coefficient (ρ) was 0.1094, positive and highly significant, indicating that the CII in one district is positively related to the CII in neighboring districts. The model’s Pseudo R² was 0.5335, demonstrating a substantial improvement in explanatory power compared with the fixed-effects model.

In SEM (Table 3), the coefficient of the day-of-week diversity index (p < 0.001) was again negative, which is consistent with the SAR model, reaffirming that a more balanced distribution of floating populations across weekdays and weekends is associated with lower CII levels (i.e., greater stability). While the other variables were not statistically significant, the change in age diversity (p < 0.1) showed a negative coefficient, indicating that adjustments toward a more balanced age composition were associated with a decrease in the CII. The spatial error coefficient (λ) was 0.1079, positive and highly significant, suggesting spatial dependence operating through the error process, whereby instability patterns in one district are linked to those in adjacent districts. The R² of the model is 0.5295, a value similar to that of the SAR model.

According to the results of the spatial dependence tests (Table 4), both the LM-lag and robust LM-lag were statistically significant at the 1% level, providing strong evidence of spatial autoregressive effects in the dependent variable. The LM-error and robust LM-error were also significant; however, when comparing the magnitudes of the test statistics and their p-values, the lag-based tests provided stronger evidence. Accordingly, the subsequent discussions and implications are presented with reference to the SAR model.

**Table 4:** Spatial dependence test results (LM and Robust LM tests).

Test	Test statistic	p-value
LM-lag	23.997	9.65e-07
Robust LM-lag	20.974	4.66e-06
LM-error	19.426	1.05e-05
Robust LM-error	16.403	5.12e-05

Note: Lagrange multiplier (LM)-lag and LM-error test for spatial lag and spatial error dependence, respectively. Robust versions account for potential misspecification when both types of dependence are present.

In summary, several variables that were insignificant in the fixed-effects model regained significance in the spatial panel models, indicating that incorporating spatial dependence among commercial districts provides important explanatory power for analyzing instability. Although temporal variations within the same district alone failed to capture these effects, the interconnections with adjacent districts revealed their influence. This finding underscores the validity of the spatial panel analysis as the core analytical framework of this study.

### Key findings and implications

This section synthesizes the results from the previous two subsections to summarize the implications of floating population diversity indicators (age, time of day, and day of the week) for the CII. The fixed-effects model analyzes temporal variation within the same commercial district but does not account for spatial dependence across districts. In contrast, the SAR and SEM models incorporate interactions with neighboring districts to estimate spatially transmitted effects. Because of this difference, some variables that were insignificant in the fixed-effects model became significant in the spatial panel models, underscoring the importance of considering spatial dependence in explaining the relationship between diversity and the CII.

First, panel and spatial panel regression analyses confirmed that for certain variables, diversity indicators and their changes preceded the prediction of the CII. In particular, the day-of-week diversity indicator consistently shows a negative coefficient across all models, indicating that more balanced distributions of weekday and weekend floating populations are associated with a subsequent reduction in the CII. In addition, changes in age diversity exhibit a negative effect at the 10% significance level, suggesting that shifts toward a more balanced age distribution are related to the mitigation of future instability.

Second, these findings suggest that both the diversity indicators of the floating population and the direction of their changes can serve as important signals for early diagnosis of district-level instability. Higher values of the day-of-week diversity indicator were associated with lower CII levels (greater stability), whereas more balanced age diversity contributed to sustaining long-term demand. Conversely, increasing the concentration during specific periods or days might elevate instability risk, underscoring the need for strategies to mitigate such tendencies. For instance, policies aimed at revitalizing commercial districts could reduce weekday–weekend or time-of-day concentrations and implement programs that attract a broader range of age groups. Moreover, as shown in the spatial panel regression results, policy design should account for not only individual districts, but also inter-district linkages, recognizing that instability in one area can spill over to its neighbors.

From a theoretical perspective, this study extends prior research by integrating floating population diversity and its changes into an analysis of the CII, while employing both panel and spatial panel regression models to verify spatial interaction effects. By considering not only temporal changes but also spatial transmission mechanisms, this study enhances the precision of the stability analysis for commercial districts. From a policy perspective, these findings provide empirical evidence that managing diversity within commercial districts can mitigate the CII, thereby offering a foundation for strategies that promote local economic stability and strengthen district resilience.

## Discussion and conclusion

This study aimed to diagnose commercial-district instability in business openings and closures within the broader context of population decline and urban restructuring. To this end, we developed the CII based on the standardized difference between openings and closures, along with diversity indices derived from the entropy of the floating population by age, time of day, and day of the week. These indices are applied to quarterly panel data at the commercial district level in Seoul and analyzed using spatial panel regression models (SAR and SEM). This integrated approach provides a new analytical framework for comprehensively understanding the structural dynamics of commercial districts and offers insights that can contribute to urban planning and district-level policy design.

The analysis revealed that the CII in Seoul was significantly shaped by the spatial dependence on adjacent areas. The positive spatial coefficients indicate that the instability is not confined to a single district but tends to diffuse to neighboring districts, highlighting its relevance to the stability of the broader urban commercial network. Among the floating population diversity indices, day-of-week diversity consistently exhibited a negative association with the CII. Districts with more evenly distributed visits across weekdays and weekends showed lower levels of instability, suggesting that a stable rhythm of consumption underpins commercial stability. In addition, changes in age diversity displayed a modest negative effect, implying that a greater intergenerational balance may help mitigate instability risks. Collectively, these findings suggest that the CII cannot be fully explained by openings and closures alone but is partly driven by structural factors such as the balance and diversity of consumption patterns.

These patterns align with our theoretical expectation that diversity spreads risk across segments and time. In particular, higher day-of-week diversity is associated with lower subsequent CII, indicating that temporal diversification buffers turnover shocks, while changes in age diversity demonstrate a modest negative association consistent with intergenerational risk-spreading. Collectively, these results substantiate the use of entropy-based diversity as an early-warning input to commercial stability.

This study makes a meaningful contribution by moving beyond the limitations of prior commercial district research, which has often relied on static indicators, such as store counts, business type distributions, and sales volume. First, it operationalizes commercial-district instability via the CII—a standardized relative deviation between openings and closures—providing a measurement framework comparable across time and space. Second, it systematically incorporates floating-population diversity (age, day-of-week, time-of-day) as an explanatory factor for the CII, empirically identifying how multidimensional compositional structures relate to instability. Third, by applying SAR and SEM models, it embeds spatial dependence into the analysis and verifies the spatial transmission of instability across commercial districts, rendering the spatial context explicit in the study of commercial dynamics.

These findings have important policy implications for future studies. The CII can serve as a proactive diagnostic tool to detect structural risks in commercial districts earlier and more effectively than conventional indicators, such as the number of establishments or sales volume. Designating areas with simultaneously high levels of business openings and closures as early-warning zones enables timely and preventive intervention. Furthermore, consistent evidence that weekday and age diversity enhance stability underscores the need for targeted policy actions in districts heavily skewed toward specific days or generations. For example, expanding weekday-oriented programs in areas with insufficient weekday activity, or introducing facilities that attract multiple generations in districts dominated by a single age group, could foster a more balanced consumer base. Finally, the finding that the instability spread across adjacent commercial districts highlights the necessity of transitioning from isolated district-level management to comprehensive region-wide governance. This shift can be particularly effective when combined with broader urban planning frameworks, such as neighborhood-scale planning or commercial network strategies.

However, this study has several limitations. First, the analysis was based on aggregated data at specific time periods and at the commercial district level, which might not fully capture long-term trends or store-level characteristics. Second, external factors such as business cycle fluctuations, the rise of online consumption, and policy interventions were not directly incorporated. Finally, although the CII—a standardized relative deviation of openings and closures—effectively diagnoses structural risk, it does not indicate whether districts that stabilize after periods of instability subsequently progress toward growth or decline.

Future research should address these limitations by examining the trajectories of commercial districts that have experienced high CII and subsequently stabilized, and by investigating how contextual factors such as demographic structure, land use, and policy interventions shape district resilience and sustainability. This would allow the CII to be understood not merely as a risk factor but as part of a dynamic process that encompasses the potential for recovery and growth.
